# Distinct Cytokine Landscapes Induced by Influenza a Virus, RSV, and SARS-CoV-2 in Older Adults (65+) Using an Ex Vivo Whole Blood Stimulation Model

**DOI:** 10.3390/pathogens15020139

**Published:** 2026-01-27

**Authors:** Annapina Palmieri, Ilaria Schiavoni, Eleonora Olivetta, Pasqualina Leone, Alessandra Fallucca, Anita Muglia, Angelo Carfì, Antonella Di Paola, Graziano Onder, Giorgio Fedele

**Affiliations:** 1 Department of Cardiovascular, Endocrine-Metabolic Diseases and Aging, Istituto Superiore di Sanità, 00161 Rome, Italy; annapina.palmieri@iss.it; 2 VPD—Reference Labs Unit, Department of Infectious Diseases, Istituto Superiore di Sanità, Viale Regina Elena 299, 00161 Rome, Italyanita.muglia@guest.iss.it (A.M.); 3 National Center for Global Health, Istituto Superiore di Sanità, 00161 Rome, Italy; 4 Department of Health Promotion, Maternal and Infant Care, Internal Medicine and Medical Specialties (PROMISE) “G. D’Alessandro”, University of Palermo, 90127 Palermo, Italy; 5 National PhD Programme in One Health Approaches to Infectious Diseases and Life Science Research, Department of Public Health, Experimental and Forensic Medicine, University of Pavia, 20171 Pavia, Italy; 6 Fondazione Policlinico Universitario “Agostino Gemelli” IRCCS, 00168 Rome, Italy; 7 Faculty of Medicine and Surgery, Università Cattolica del Sacro Cuore, 00168 Rome, Italy

**Keywords:** whole-blood, cytokine gene expression, older adults, influenza virus, respiratory syncytial virus, SARS-CoV-2

## Abstract

Exaggerated immune responses to respiratory viruses may contribute to increased morbidity in older adults. To investigate virus-specific immune activation in this population, we developed an ex vivo whole blood stimulation model using samples from 30 healthy individuals aged ≥65 years. Whole blood was stimulated with UV-inactivated influenza A virus (IAV), respiratory syncytial virus (RSV), and SARS-CoV-2, and the expression of 22 immune-related genes was assessed by quantitative RT-PCR array. All three viruses elicited responses with marked variability across individuals, as well as differences in the magnitude and distribution of cytokine expression across stimuli. RSV stimulation was associated with relatively higher expression of inflammatory mediators, while IAV and SARS-CoV-2 induced greater expression of Type I interferon. SARS-CoV-2 also led to an increased expression of regulatory cytokines. Although individual responses varied, correlation analysis indicated coordinated gene expression within functional categories, and Uniform Manifold Approximation and Projection (UMAP) showed distinct grouping of cytokine responses by virus and function. These findings describe differential immune mRNA expression profiles in response to viral stimuli in older adults and may support future studies aimed at understanding age-related differences in host–virus interactions.

## 1. Introduction

Respiratory viruses remain a leading cause of severe illness and death worldwide, especially among older adults. Influenza A virus (IAV) alone causes 290,000–650,000 deaths globally each year, with 90% of fatalities occurring in adults aged ≥65 years [1]. Similarly, respiratory syncytial virus (RSV) infection in hospitalized older adults is associated with morbidity and mortality rates comparable to or even higher than those of influenza [2], and SARS-CoV-2 mortality can reach 15–20% in patients over 80 years old [3].

Host immune responses to viral infections are key determinants of disease outcomes in older adults. In these subjects, respiratory viral infections are frequently associated with increased morbidity and mortality [4,5,6]. A deeper understanding of the immune mechanisms activated by these infections in older adults is therefore urgently needed.

Upon respiratory tract infection, the innate immune system is rapidly activated to control viral replication and recruit immune cells. A complex network of reciprocally regulated soluble mediators then participates in orchestrating the overall immune response. Proinflammatory cytokines are produced to contain the pathogen, interferons (IFNs) and other antiviral mediators suppress further viral replication, and regulatory cytokines are activated to mitigate excessive inflammation and prevent tissue damage [7,8,9,10,11]. Despite their shared capacity to engage these innate and regulatory pathways, IAV, RSV, and SARS-CoV-2 each drive distinct cytokine landscapes. Recent comparative analyses indicate that COVID-19 patients often exhibit weaker overall immune activation, characterized by lower enrichment of antiviral signatures and dysregulated type I IFN responses, than influenza or RSV cases [12,13,14,15,16,17]. These virus-specific signatures may be further modulated by age-related changes in immune function, such as immunosenescence and inflammaging, leading to chronic inflammation and long-term damage [18,19,20,21].

This study aims to compare the differential immune gene expression profiles induced by IAV, RSV, and SARS-CoV-2 in older adults using an ex vivo whole blood stimulation model. By stimulating whole blood from uninfected adults aged 65 and older with UV-inactivated viruses, we aimed to characterize the differential gene expression patterns in response to each virus. Defining these immune signatures in the older adult population will guide the development of targeted immunomodulatory strategies for this high-risk population.

## 2. Methods

### 2.1. Study Design and Participants

Forty-four uninfected older adults (aged 65 years and older) were enrolled from the Geriatric Clinic at Agostino Gemelli University Hospital in Rome, Italy, after providing written informed consent. Exclusion criteria included any ongoing infections as well as conditions such as immunodeficiency or immunosuppression, autoimmune diseases, active cancer, diabetes, chronic obstructive pulmonary disease, obesity, and coronary artery disease. Information on demographics, previous vaccinations, and history of respiratory infections was gathered from each participant. Medical vulnerability was assessed using Basic and Instrumental Activities of Daily Living and Mini Mental State Examination scores [22]. Blood samples were successfully cultured from only 32 participants, due to technical difficulties or issues related to sample quality. Since RNA expression data was missing for 1 subject, and 1 subject was excluded due to a recent COVID-19 infection, the final sample consisted of 30 participants (Supplementary Figure S1). Table 1 presents the demographic characteristics of the study sample.

### 2.2. Inactivated Virus Preparation

Ultraviolet (UV)-inactivated viral preparations were generated to render the viruses non-infectious while preserving their antigenic integrity for subsequent stimulation assays. Specifically, RSV Type A2 strain (ATCC VR-1540), Influenza A (clinical isolate A/H1N1); SARS-CoV-2 (clinical isolate belonging to the B lineage, Wuhan strain) were propagated to obtain viral stocks and titers were determined by standard plaque assays. Aliquots of each virus were placed in an open-lidded container inside a BH-EN-2004 Class II Type A2 biosafety cabinet (Faster, Cornaredo (MI), Italy) and exposed to the cabinet’s integrated 18 W UV-C lamp (253.7 nm) for a fixed duration of 2 h. Viral inactivation was confirmed by the absence of viral genome amplification via RT-qPCR on the supernatant of cells exposed to the UV-treated virus preparations.

### 2.3. Diluted Whole Blood Cultures

Venous blood (2.0–5.0 mL) was drawn by venipuncture into heparinized tubes from each consenting participant. Within 30 min of collection, blood was gently mixed 1:1 (*v*/*v*) with pre-warmed RPMI-1640 medium supplemented with 2 mM L-glutamine, 100 U/mL penicillin, 100 µg/mL streptomycin, and 10 mM HEPES (complete RPMI). All media and supplements were purchased from Gibco (Thermo Fisher Scientific, Waltham, MA, USA). Diluted blood was dispensed at a final volume of 0.6 mL into sterile 12 × 75 mm tubes. Each sample was stimulated with ultraviolet-inactivated viral preparations at an MOI of 1 based on preliminary experiments. Control tubes were set up adding sterile phosphate-buffered saline (PBS). The blood cultures were incubated for 24 h at 37 °C, 5% CO_2_. The choice of stimulation length was based on previous whole blood and PBMC stimulation studies, where incubation periods of 16–24 h have been shown to provide measurable and reproducible cytokine gene expression. Since our experiments used UV-inactivated viruses, replication-dependent effects were absent, and our results therefore capture early transcriptional responses to viral antigen exposure in a standardized ex vivo setting. Experiments were performed without duplicates to maximize the number of distinct donors and conditions, given the limited sample volume and high cost of reagents.

### 2.4. Gene Expression Analysis

Total RNA was extracted from the blood cultures after 24 h of diluted whole blood stimulation using the total RNA Purification Kit (Norgen Biotek Corp., Thorold, ON, Canada). Gene expression was analyzed using TaqMan Array plates (Thermo Fisher Scientific), customized to assess a broad range of genes involved in inflammation and immune responses. Quantitative real-time PCR was carried out with a Viia7 real-time PCR system (Applied Biosystems, Foster City, CA, USA). Results were normalized to GAPDH gene expression and calculated using the 2^−ΔΔCt^ method, therefore presented as fold increase relative to untreated samples.

### 2.5. Statistical Analysis

Descriptive statistics were used to summarize cytokine mRNA expression profiles. Mann–Whitney tests assessed differences in cytokine responses between male and female participants (Table 1). Data processing and statistical analyses were conducted using Python (version 3.11.13) with standard scientific libraries including NumPy, pandas, SciPy, and scikit-learn. Gene expression values, calculated as fold changes using the 2^−ΔΔCt^ method, were log-transformed when necessary to improve normality of distributions. Normality of data was assessed using the Shapiro–Wilk test implemented in SciPy. Due to non-normal distribution and limited sample size, non-parametric tests were predominantly applied. Differences in gene expression and cytokine levels across the three viral stimulations (Influenza A, RSV, SARS-CoV-2) were evaluated using the Kruskal–Wallis H-test. Pairwise correlations among cytokine expression levels were assessed with Spearman correlation coefficients and visualized with seaborn heatmaps to identify coordinated expression patterns. A *p*-value for multiple testing was adjusted with the Benjamini–Hochberg false discovery rate (FDR) method, considering a significance level of *p*  <  0.05. For dimensionality reduction and clustering of cytokines by functional categories, Uniform Manifold Approximation and Projection (UMAP) was applied using the Python implementation from the umap-learn library. Statistical significance was set at *p* < 0.05 for all tests.

## 3. Results

### 3.1. Study Sample

The study’s final sample comprised 30 older adults, with a mean age of 74.6 years (Min–Max: 65–86). The sample was composed of 12 males (mean age 74.9 years, Min–Max: 65–86) and 18 females (mean age 74.3 years, Min–Max: 65–86), with no significant age difference between sexes (*p* = 0.8).

Regarding clinical characteristics, 27% of the overall sample had osteoporosis, 13% had hypertension, and 20% had medical vulnerability, determined by a multidimensional assessment which evaluates a comprehensive range of physical, psychological, and functional factors. A substantial proportion of the participants (77%) were vaccinated against influenza. Twenty-nine participants (97%) were vaccinated against SARS-CoV-2. Among them, 15 (50% of the total cohort) had received four doses, 13 (43%) had received three doses, and one (3.3%) had received two doses. One participant (3.3%) was unvaccinated. No significant differences were observed between sexes for any of these clinical variables or vaccination statuses (Table 1).

### 3.2. Cytokine mRNA Profiles Following Stimulation with IAV, RSV, and SARS-CoV-2

To characterize immune responses elicited by respiratory viruses in older adults, diluted whole blood cultures from donors aged ≥65 years were stimulated 24 h with UV-inactivated Influenza A virus (IAV), RSV, and SARS-CoV-2. The transcriptional levels of 21 immune-related genes were measured by customized RT-PCR arrays, alongside CD38, a surface receptor implicated in inflammation and immune regulation. Cytokines and chemokines were grouped functionally into early inflammatory (TNFα, IL-6, IL-1β, CCL2, CCL3, CCL5, CXCL10), chronic inflammatory (IL-12A, IL-17A, IL-5, IL-13, IL-15, IL-18, IFNγ), suppressive (IL-10, IL-1RN, CTLA4), and antiviral (IFNα1, IFNβ1, IFNλ1, ISG15) categories.

**Figure 1:**
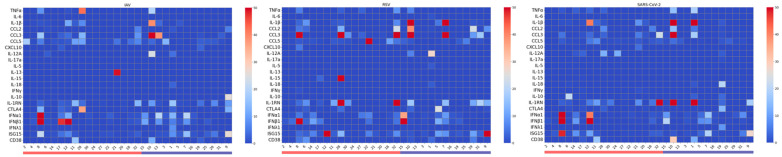
Heatmaps of cytokine gene expression in ex vivo whole-blood cultures from older adults (≥65 years) stimulated with UV-inactivated respiratory viruses. Heatmaps depict the mean fold increase in mRNA levels of 22 cytokines and chemokines relative to unstimulated controls for each donor after 24 h incubation with Influenza A/H1N1, RSV type A2, or SARS-CoV-2. Rows list individual cytokine genes (left axis), and columns correspond to donors grouped females (red bracket) and males (blue bracket). The color-coded scale bar on the right indicates the mRNA expression levels, ranging from 0 (blue, minimum) to 50 (red, maximum).

**Figure 2:**
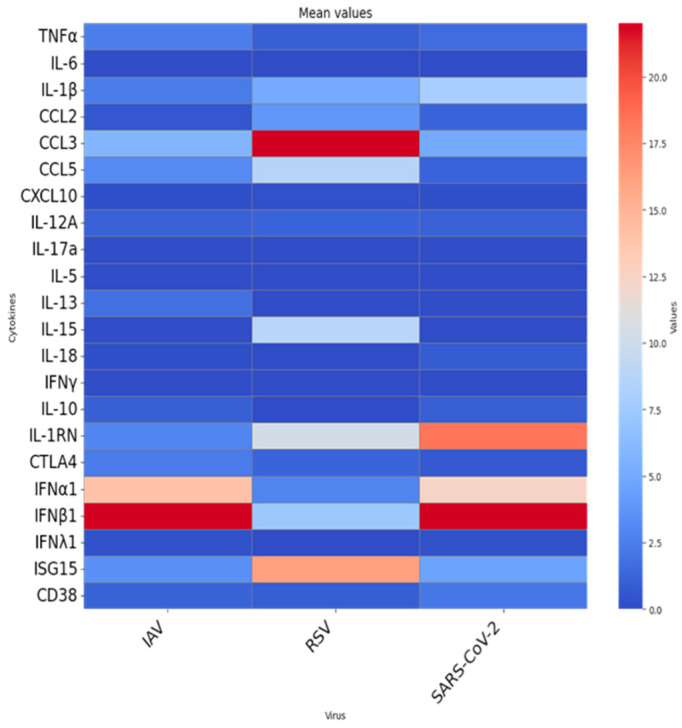
Mean fold-change in cytokine and immune-regulatory gene expression in ex vivo whole-blood cultures from older adults (≥65 years) stimulated with UV-inactivated respiratory viruses. Heatmap depict the mean fold-change in gene expression in ex vivo whole-blood cultures stimulated with inactivated influenza A virus (IAV), RSV, or SARS-CoV-2. Each column shows the average 24-h fold induction of 22 genes in 30 blood donors. Rows list individual gene targets.

As shown in Figure 1, stimulation with each virus elicited distinct and virus-specific cytokine mRNA expression profiles, with substantial inter-individual variability across donors. Despite this heterogeneity, virus-specific patterns emerged. RSV stimulation triggered upregulation of early inflammatory chemokines CCL2, CCL3, and CCL5. In contrast, IAV and SARS-CoV-2 elicited more moderate inflammatory responses, suggesting differential early immune activation mechanisms among viruses. Figure 2 illustrates the mean fold-change in gene expression for each cytokine. Although most comparisons did not reach statistical significance, the expression patterns revealed relevant differences. IAV and SARS-CoV-2 both induced relatively high levels of type I interferons (IFNα1, IFNβ1), while RSV stimulation resulted in elevated ISG15 expression. When we tested for associations between participants’ characteristics (sex, vaccination status, recent COVID-19 diagnosis, and clinical conditions) and virus-induced cytokine expression, no statistically significant differences were observed.

### 3.3. Functional Group-Specific Responses

As shown, in our ex vivo model, early inflammatory genes were strongly induced by RSV, moderately by SARS-CoV-2, and to a lesser extent by IAV. Chronic inflammatory cytokines showed modest induction overall. Among three viruses, RSV showed the highest mean levels of IL-15, suggesting a potential involvement in T-cell activation and survival, consistent with a more sustained cellular immune engagement. Mean levels of the suppressive cytokine IL-1RN were higher for SARS-CoV-2 compared to IAV and RSV, suggesting an immunomodulatory attempt to limit excessive inflammation. CTLA4 showed low levels in all three viruses. Antiviral gene expression revealed virus-specific trends. IAV and SARS-CoV-2 displayed higher mean expression of type I IFN genes (IFNα1, IFNβ1), whereas RSV elicited low expression of these genes but induced strong ISG15 expression, suggesting activation of alternative antiviral signaling pathways potentially independent of classic interferon cascades.

**Figure 3:**
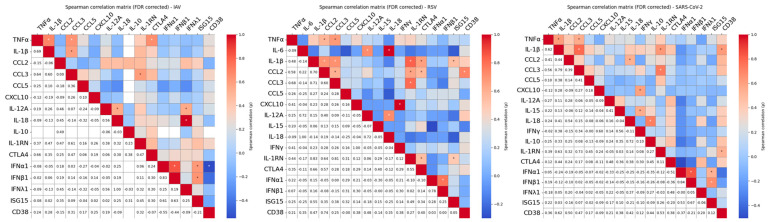
Spearman multiple pairwise correlations among cytokines following whole blood stimulation with different respiratory viruses. Heatmaps displays pairwise Spearman correlation coefficients (ρ) between cytokine levels in response to stimulation with UV-inactivated Influenza A virus (IAV), Respiratory Syncytial Virus (RSV), or SARS-CoV-2. The upper triangle displays color-coded correlation coefficients (ρ) based on strength and direction (red = positive, blue = negative). Statistically significant correlations (|ρ| > 0.3 and FDR-corrected *p* < 0.05) are marked with an asterisk (*). The lower triangle reports the corresponding numerical values. This dual representation highlights both the magnitude and the significance of cytokine associations. Only cytokines with non-zero variance across all samples are included.

Pairwise Spearman correlation analysis was performed to investigate coordinated cytokine responses. As shown in Figure 3, inflammatory cytokine genes showed strong positive correlations across stimuli, indicating a coordinated inflammatory network. Notably, IL-1RN also positively correlated with inflammatory mediators, suggesting the activation of compensatory homeostatic mechanisms. Chronic inflammatory cytokines, particularly in response to RSV, correlated with early inflammatory genes, supporting the idea of a linked acute-to-chronic inflammatory transition specific to RSV stimulation. CD38 expression, a marker of immune cell activation, correlated positively with pro-inflammatory gene expression following SARS-CoV-2 and RSV stimulation (Figure 3), reinforcing its role as a potential amplifier of virus-induced inflammation.

### 3.4. Comparative Analysis of Virus-Induced mRNA Expression Profiles

**Figure 4:**
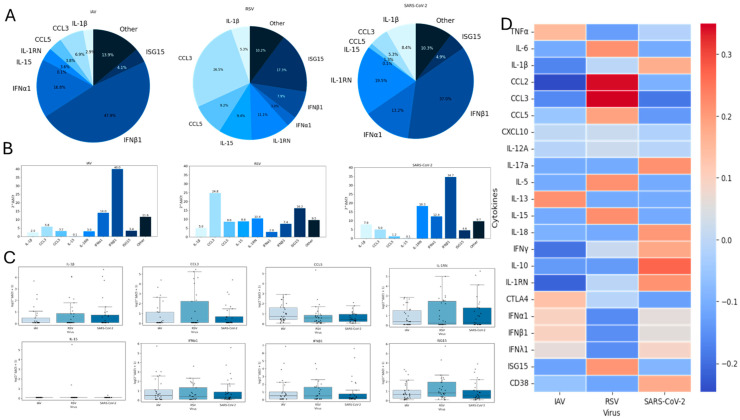
Comparative analysis of virus-induced immune gene expression. (**A**). Pie chart showing the relative contribution of the eight cytokines with the highest expression levels in our dataset. (**B**). Bar charts showing the 2^ΔΔCt^ values of these eight cytokines across virus stimulations, representing fold change relative to unstimulated controls. (**C**). Box plots showing the distribution of log-transformed fold changes [log(2^ΔΔCt^ + 1)] for the same eight cytokines, enabling comparison of response magnitude and variability across donors. (**D**). Heatmap of all 22 cytokines, scaled by row z-score, showing relative patterns of induction across viruses and highlighting coordinated functional clusters within cytokine groups. Statistical significance was determined using the Kruskal–Wallis test (*p* < 0.05).

To better define virus-specific immune activation patterns, we performed a comparative analysis across the three viral stimuli (Figure 4). Analysis of the eight most prevalent cytokines revealed a shared early-stage antiviral profile for IAV and SARS-CoV-2, characterized by antiviral rather than inflammatory mediators (with the notable exception of higher IL-1RN induction by SARS-CoV-2), whereas RSV stimulation elicited lower antiviral cytokine levels and higher expression of inflammatory chemokines, highlighting its distinct, more inflammatory signature (Figure 4A,B). Boxplots in Figure 4C (log-transformed values) and Table 2 (median, Q1–Q3, mean, SD, range) summarize the distribution of cytokine levels induced by each viral stimulus (IAV, RSV and SARS-CoV-2) and suggest the existence of distinct response patterns; however, no statistically significant differences were observed between groups. Overall, most cytokines showed low median values, but substantial variation in upper ranges allowed identification of virus-specific trends. RSV stimulation elicited the strongest inflammatory response, particularly evident for chemokines such as CCL3, and CCL5, where both mean levels and interquartile ranges were markedly higher than in IAV or SARS-CoV-2. For instance, RSV induced a mean of 24.8-fold increase for CCL3 mRNA expression versus 5.8 (IAV) and 5.0 (SARS-CoV-2), with a maximum exceeding 230, highlighting the strong pro-inflammatory skewing. In contrast, IAV and SARS-CoV-2 stimulated higher levels of antiviral mediators, including IFNα1, IFNβ1, and ISG15, with mean values reaching up to a fold increase in gene expression of 39 (IAV, IFNβ1) and 34.7 (SARS-CoV-2, IFNβ1) (Table 2). These distributions were characterized by wide ranges and high standard deviations, indicating heterogeneous activation. RSV, in comparison, showed consistently lower values in these antiviral mediators. Of note, IL1RN, a key anti-inflammatory regulator, was markedly elevated in response to SARS-CoV-2, with a mean of 18.3 and a maximum of 258.7 gene expression fold increase, compared to 2.98 for IAV and 10.4 for RSV. This suggests a virus-specific compensatory mechanism aimed at controlling excessive inflammation. Several cytokines such as IL6, IL5, IL13, IL17a, and IFNγ were consistently low across all viral conditions, as indicated by identical medians (0.10, Table 2) and minimal spread, reflecting their limited activation in this experimental setting. These data reinforce the idea that RSV triggers a more inflammatory milieu, while IAV and SARS-CoV-2 preferentially induce antiviral responses, with SARS-CoV-2 also showing signs of stronger immune regulation through IL1RN. This landscape is supported when cytokines are scaled to row z-score (Figure 4D). The resulting heatmap shows gene expression data reflecting their lower (blue shades) and higher (red shades) values below and above the mean across all samples for each cytokine across virus groups (IAV, RSV, SARS-CoV-2). Notably, RSV induces upregulation of several inflammatory mediators, including CCL2, CCL3, and CCL5, which are elevated compared to the other viruses. SARS-CoV-2 elicits a broader response including chronic inflammatory cytokines, along with an increase in suppressor cytokines, indicative of a regulatory feedback loop. IAV shows a milder and more selective activation pattern with the involvement of Type I IFNs. Overall, these results suggest the existence of distinct cytokine response patterns; however, no statistically significant differences were observed between viruses. Such patterns may nonetheless reflect underlying differences in how each pathogen engages innate immune pathways and modulates the balance between inflammation, antiviral defense, and immune regulation.

### 3.5. Functional Clustering by UMAP Analysis

**Figure 5:**
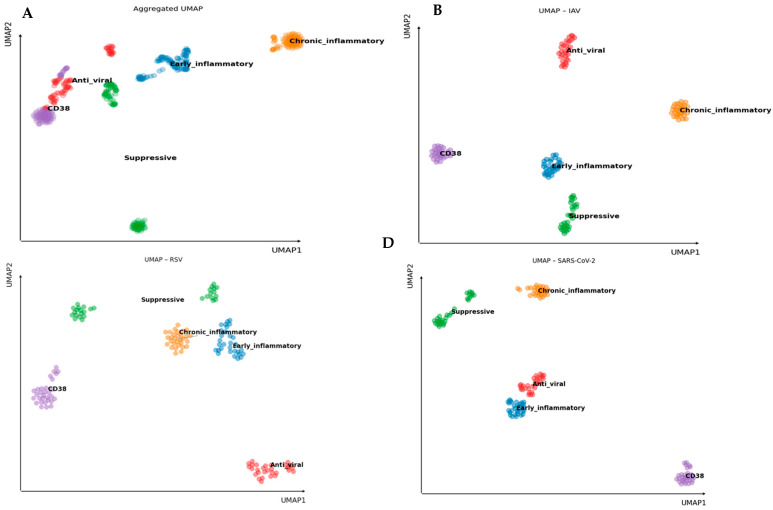
Functional Clustering of Virus-Induced Cytokine Profiles by UMAP Analysis. Uniform Manifold Approximation and Projection (UMAP) dimensionality reduction was applied to 22 genes grouped into five functional categories: Early inflammatory, Chronic inflammatory, Suppressive, Anti-viral, and CD38. UMAP was performed on fold-change values calculated relative to unstimulated baseline Ct values for each donor. (**A**). Aggregated UMAP showing the global cytokine signature distribution across all after stimulation with UV-inactivated viruses. (**B**–**D**). Virus-specific UMAP plots for (**B**) IAV, (**C**) RSV, and (**D**) SARS-CoV-2. Functional clusters remain clearly defined across all infections, indicating coordinated co-expression within functional groups despite virus-specific variations in immune response profiles.

To investigate whether the immune response to the three viruses resulted in distinct functional patterns, we performed Uniform Manifold Approximation and Projection (UMAP) dimensionality reduction to cytokine expression data, with genes grouped into five functional categories: early inflammatory, chronic inflammatory, suppressive, antiviral, and CD38. This approach enabled visualization of the overall structure and functional organization of the immune response landscape elicited by each viral stimulus (Figure 5).

As shown in Figure 5A, UMAP analysis of the combined data from all viruses reveals clear separation of cytokine clusters based on their functional categories. These spatially well-defined clusters indicate consistent and distinguishable expression profiles within each group, supporting the notion that cytokines within a functional category act in a coordinated and biologically meaningful manner.

Further exploration of virus-specific responses (Figure 5B–D) demonstrates that these functional clusters remain distinct across IAV, RSV, and SARS-CoV-2. Although the spatial arrangement of clusters varies slightly depending on the virus, reflecting subtle differences in immune activation patterns, the integrity of intra-group segregation is maintained. This suggests that cytokines within each functional category tend to be co-expressed similarly in response to each pathogen.

UMAP analysis confirms that cytokines cluster into relevant functional profiles in the immune responses of older individuals to these viral infections, highlighting distinct cytokine landscapes associated with each virus.

## 4. Discussion

In this study, we investigated the cytokine responses of older adults (≥65 years) to ex vivo stimulation with three major respiratory viruses: IAV, RSV, and SARS-CoV-2. Our findings reveal virus-specific immune signatures and inter-individual variability, underscoring the complexity of the host response in the aging population. Since our study included only individuals aged ≥65 years, these patterns describe variability within the elderly cohort and cannot be directly interpreted as age-related differences. The gene panel analyzed was designed to encompass key functional components of the host immune response. It included inflammatory cytokines and chemokines that drive and sustain inflammation and promote leukocyte recruitment; type I and III interferons together with interferon-stimulated genes that establish antiviral states; and regulatory mediators, able to limit or modulate immune activation. By incorporating genes across these axes, plus CD38 as a marker of immune activation, our analysis was designed to capture the breadth of possible responses to viral stimulation in blood from older adults.

The cytokine mRNA expression profiles demonstrated marked donor-to-donor heterogeneity, highlighting the influence of host-intrinsic factors such as genetic variability on immune responsiveness. This observation supports previous findings that genetic factors significantly shape cytokine responses to infectious stimuli [23], albeit this variation may also be influenced by environmental factors.

Despite this variability, clear patterns of virus-specific cytokine induction were noticeable. Across all three viruses, we observed positively correlated expression of pro-inflammatory genes, suggesting a coordinated activation of inflammatory pathways. This points to shared immunological circuits engaged during the early phases of viral recognition. Of particular interest was the consistent association of CD38 expression with inflammatory cytokines in response to both SARS-CoV-2 and IAV, suggesting that CD38 is involved in the regulation of virus-induced inflammation. This supports emerging evidence implicating CD38 in the regulation of inflammatory processes [24,25] and positions it as a potential therapeutic target to mitigate excessive inflammation in older adults [26].

Virus-specific immune dynamics were evident in both the strength and composition of the inflammatory response. RSV elicited a pro-inflammatory signature consistent with findings from several previous studies [27,28,29]. In particular, the induction of chemokines is critical for leukocyte recruitment and activation; their upregulation by RSV, while potentially effective in limiting viral replication, may also contribute to airway inflammation and remodeling, especially in vulnerable populations such as older adults [30]. In contrast, SARS-CoV-2 and influenza induced less pronounced inflammatory responses, indicating distinct strategies of immune engagement. SARS-CoV-2 in particular induced higher expression of suppressor cytokines, which could represent a mechanism to evade immune detection, particularly by dampening the initial activation of type I interferons [31,32]. Although our exploratory analysis did not reveal significant associations between prior vaccination and virus-specific cytokine responses, the high vaccination coverage in our cohort likely limited statistical power to detect subtle effects. Future studies with larger, stratified cohorts will be necessary to determine whether vaccine-induced immunity modulates ex vivo cytokine responses to viral stimulation.

A deeper analysis of cytokine mRNA expression profiles, assessed by the row z-score, revealed virus-specific regulatory and antiviral features. SARS-CoV-2 prominently induced IL-18 and IFN-γ, suggesting a Th1-skewed response and potential for chronic immune activation. The co-induction of IL-10 and IL-1 receptor antagonist (IL-1RN) may reflect an attempt to counterbalance inflammation and prevent excessive tissue damage. This duality underscores the immunoregulatory paradox of SARS-CoV-2: the capacity to both provoke hyperinflammation and induce immunosuppression [33]. This imbalance may be envisaged as a contributor to disease severity and immune dysfunction in COVID-19, particularly among older individuals. RSV induces IL-15, a cytokine involved in the survival and activation of T and NK cells [34]. The robust IL-15 induction observed in our study highlights its potential significance, as IL-15 is known to be critical for the maintenance and survival of memory T cells [35]. While our findings do not directly assess long-term effects, they suggest that IL-15 may have a role in shaping long-term immunity to RSV and could be a factor in the immune pathology associated with post-infectious sequelae such as recurrent wheezing or asthma-like symptoms. Concerning the antiviral response observed systemically in the blood, both IAV and SARS-CoV-2 evoked IFNα and IFNβ, indicative of a classical antiviral response. However, neither virus significantly induced IFNλ in the blood, which aligns with its predominant role at mucosal surfaces rather than in systemic circulation [36]. Consistent with the existing literature [37,38], our findings show that RSV upregulates ISG15, an interferon-stimulated gene involved in antiviral responses. This is notable as it occurred without a strong induction of Type I IFN. This suggests that RSV may activate alternative innate immune pathways, such as RIG-I or MDA5 signaling, to mount an antiviral response while minimizing overt inflammation [39]. This strategy may allow for sustained immune activity without triggering excessive immune-mediated tissue injury. Noteworthy is that this experimental approach was selected to capture early, antigen-driven transcriptional responses in a simplified system while minimizing confounding effects associated with viral replication and cytopathic damage. Nevertheless, replication-associated pathways likely influence immune activation, and direct comparisons with live viruses in biosafe environments will be needed to confirm the relevance of our findings.

Taken together, these virus-specific immune profiles reflect distinct host–pathogen interactions and immune evasion strategies, with implications for the pathogenesis and clinical course of infection in older adults. Although direct comparisons with younger adults were not performed in this study, the virus-specific immune signatures we observe may reflect age-related remodeling of innate and adaptive antiviral responses, including dampened interferon induction and selective chemokine upregulation, in line with the broader concept of immunosenescence, in which aging is associated with reduced activation of antiviral and inflammatory pathways and altered cytokine and chemokine profiles [40,41].

Our study has several limitations. First, the use of ex vivo whole blood stimulation captures the systemic immune potential but does not fully recapitulate the tissue-specific immune responses of the respiratory tract, including localized epithelial–immune crosstalk. Furthermore, this study was restricted to healthy older adults; therefore, our findings may not be fully generalizable to older adults with comorbidities or different health statuses. Second, the use of a single stimulation time point is a critical factor in interpreting our findings. The kinetics of cytokine induction vary substantially across respiratory viruses: influenza A (IAV) elicits a rapid interferon and inflammatory response, often within the first 24–48 h [42]; RSV tends to induce more delayed and prolonged responses [43]; while SARS-CoV-2 is characterized by an initially attenuated or delayed interferon response followed by strong pro-inflammatory cytokine production at later stages [40]. Thus, the mRNA expression profiles observed here may reflect not only intrinsic differences in immune pathways but also distinct temporal stages of host responses. Future studies including multiple stimulation time points will be important to disentangle differences in kinetics from differences in magnitude of the response. Moreover, some donor samples show minimal or absent expression changes across all conditions and suboptimal stimulation, donor variability or RNA degradation might contribute. Third, the sample size, while adequate to detect major trends, limits the statistical power to detect more subtle effects of demographic and clinical variables such as sex, comorbidities, and prior infection history. Fourth, the observational nature of cytokine profiling precludes mechanistic insights into causal pathways. Finally, our stimulations were performed with UV-killed viruses rather than live viruses. While UV-inactivated viruses can effectively stimulate key immune pathways, they lack the ability to replicate and induce cellular stress responses integral to natural infection. This may lead to differences in the host immune response compared to a full viral infection. Furthermore, although we observed associations involving CD38, functional validation of its role in viral inflammation was beyond the scope of the present study.

In conclusion, this study demonstrates that older adults exhibit distinct and virus-specific blood cytokine responses to major respiratory viruses, shaped by both intrinsic factors and pathogen characteristics. RSV response is marked by inflammatory genes’ activation with the potential for long-term immune activation, while SARS-CoV-2 exhibits features of immune evasion coupled with compensatory antiviral responses. Influenza shows a more canonical antiviral profile with moderate inflammation and effective interferon responses. The consistent correlation between CD38 and inflammatory markers highlights its role as a central modulator of virus-induced immune responses in aging hosts.

The differential cytokine landscapes observed in this study provide insights into the immune vulnerabilities of older adults to respiratory viruses. Heightened inflammation (as seen with RSV) or dysregulated immune balance (as with SARS-CoV-2) may contribute to the higher morbidity and mortality seen in this population. Immune evasion may delay viral clearance and permit persistent replication, potentially contributing to downstream pathology such as acute respiratory distress syndrome (ARDS) and long COVID. Influenza, while inducing a somewhat stronger early response than SARS-CoV-2, showed a more balanced cytokine pattern that may reflect an optimized trade-off between viral control and tissue preservation. Targeting key modulators such as CD38 could offer a strategy to temper excessive inflammation without compromising antiviral defense. Future work integrating parallel quantification of secreted cytokines will be required to validate these findings and to delineate the functional consequences of the distinct transcriptional signatures observed in elderly individuals.

Overall, these findings underscore the need for age-tailored therapeutic interventions and support the inclusion of immunomodulatory approaches in future strategies for managing respiratory infections in older adults. Future research should expand on these results by exploring the interplay between cytokine regulation and clinical outcomes in aging populations.

## Figures and Tables

**Table 1:** Demographic and clinical characteristics of the study participants.

	Whole Sample (N = 30)	Male (N = 12)	Female (N = 18)	*p*-Value
Age (year)	Mean = 74.6	Mean = 74.9	Mean = 74.3	0.8
	Min–Max = 65–86	Min–Max = 65–86	Min–Max = 65–86	
Osteoporosis	8 (27%)	0 (0%)	8 (100%)	0.02
Hypertension	4 (13%)	4 (100%)	0 (0%)	0.04
Medical Vulnerability	6 (20%)	3 (50%)	3 (50%)	0.92
Vaccination status				
Influenza vaccination (current season 2022/2023)	23 (77%)	9 (39%)	14 (61%)	0.37
Number of doses anti-SARS-CoV-2				
No vaccination	1 (3.3%)	0	1 (100%)	0.61
2 doses	1 (3.3%)	0	1 (100%)	
3 doses	13 (43.3%)	7 (54%)	6 (46%)	
4 doses	15 (50%)	5 (25%)	10 (75%)	
COVID-19 diagnosis (>1 y)	8 (27%)	3 (37%)	5 (63%)	0.65
Influenza diagnosis (<1 m)	1 (3%)	0	1 (100%)	1.00

**Table 2:** Cytokine levels of eight prevailing cytokines and associated boxplot comparisons.

Cytokine	Virus	Median (Q1, Q3)	Mean (SD)	Min–Max	*p*-Value
**IL6**	IAV	0.10 (0.10, 0.10)	0.10 (0.00)	0.10–0.10	0.36
	RSV	0.10 (0.10, 0.10)	0.131 (0.17)	0.10–0.10	
	SARS-CoV-2	0.10 (0.10, 0.10)	0.10 (0.00)	0.10–0.10	
**CCL3**	IAV	0.10 (0.10, 2.09)	58.16 (16.40)	0.10–80.60	0.89
	RSV	0.10 (0.10, 8.36)	24.81 (58.87)	0.10–236.96	
	SARS-CoV-2	0.10 (0.10, 0.10)	5.01 (15.76)	0.10–82.19	
**CCL5**	IAV	1.141 (0.56, 3.94)	3.16 (4.41)	0.10–17.62	0.08
	RSV	0.829 (0.21, 1.40)	8.62 (58.87)	0.10–211.84	
	SARS-CoV-2	0.10 (0.10, 0.10)	1.24 (1.64)	0.10–7.29	
**IL5**	IAV	0.10 (0.10, 0.10)	0.10 (0.00)	0.10–0.10	0.36
	RSV	0.10 (0.10, 0.10)	0.15 (0.00)	0.10–0.10	
	SARS-CoV-2	0.10 (0.10, 0.10)	0.10 (0.00)	0.10–0.10	
**IL1RN**	IAV	0.48 (0.10, 3.79)	2.98 (4.76)	0.10–16.30	0.97
	RSV	0.28 (0.10, 11.16)	10.39 (28.25)	0.10–149.32	
	SARS-CoV-2	0.30 (0.10, 3.79)	18.31 (55.45)	0.10–258.66	
**IFN** **α1**	IAV	0.665 (0.10, 2.032)	14.03 (58.38)	0.10–316.11	0.79
	RSV	0.506 (0.10, 2.80)	2.84 (6.88)	0.10–36.28	
	SARS-CoV-2	0.39 (0.17, 1.41)	12.43(49.95)	0.10–268.71	
**IFN** **β1**	IAV	0.68(0.10, 1.74)	39.98 (177.97)	0.10–958.59	0.93
	RSV	0.64 (0.10, 4.04)	7.35 (21.16)	0.10–104.88	
	SARS-CoV-2	0.29 (0.10, 0.10)	34.75 (140.02)	0.10–730.59	
**ISG15**	IAV	0.93 (0.27, 2.02)	3.44 (6.42)	0.10–26.33	0.25
	RSV	1.28 (0.53, 6.17)	16.21 (60.21)	0.10–326.92	
	SARS-CoV-2	0.67 (0.26, 2.02)	4.59 (10.23)	0.10–48.39	

Data are presented as median (first quartile [Q1] = 25th percentile, third quartile [Q3] = 75th percentile), mean (standard deviation), and range (min–max). These numerical data correspond to the boxplots shown in Figure 4C, enabling visual comparison of cytokine distributions across the three viral groups.

## Data Availability

The datasets used and/or analyzed during the current study are available from the corresponding author on reasonable request.

## Supplementary Materials

The following supporting information can be downloaded at: https://www.mdpi.com/article/10.3390/pathogens15020139/s1, Supplementary Figure S1. Participant enrollment and selection. Flowchart depicting recruitment, screening, and exclusion criteria for study participants. A total of 44 uninfected older adults (≥65 years) were enrolled at the Geriatric Clinic in Rome. Twelve participants were excluded due to blood culture failure. Of the 32 eligible participants with successful blood cultures, one was excluded due to missing RNA data and one due to recent COVID-19 infection, resulting in a final study sample of 30 participants.
