# Supplementary material for: Distinct Cytokine Landscapes Induced by Influenza a Virus, RSV, and SARS-CoV-2 in Older Adults (65+) Using an Ex Vivo Whole Blood Stimulation Model
Source: Pathogens. 2026 Jan 27;15(2):139. doi: 10.3390/pathogens15020139 (PMC12943484; doi:10.3390/pathogens15020139)
Supplement: Supplementary file 1 [file pathogens-15-00139-s001.zip › pathogens-4073767-supplementary.pdf]

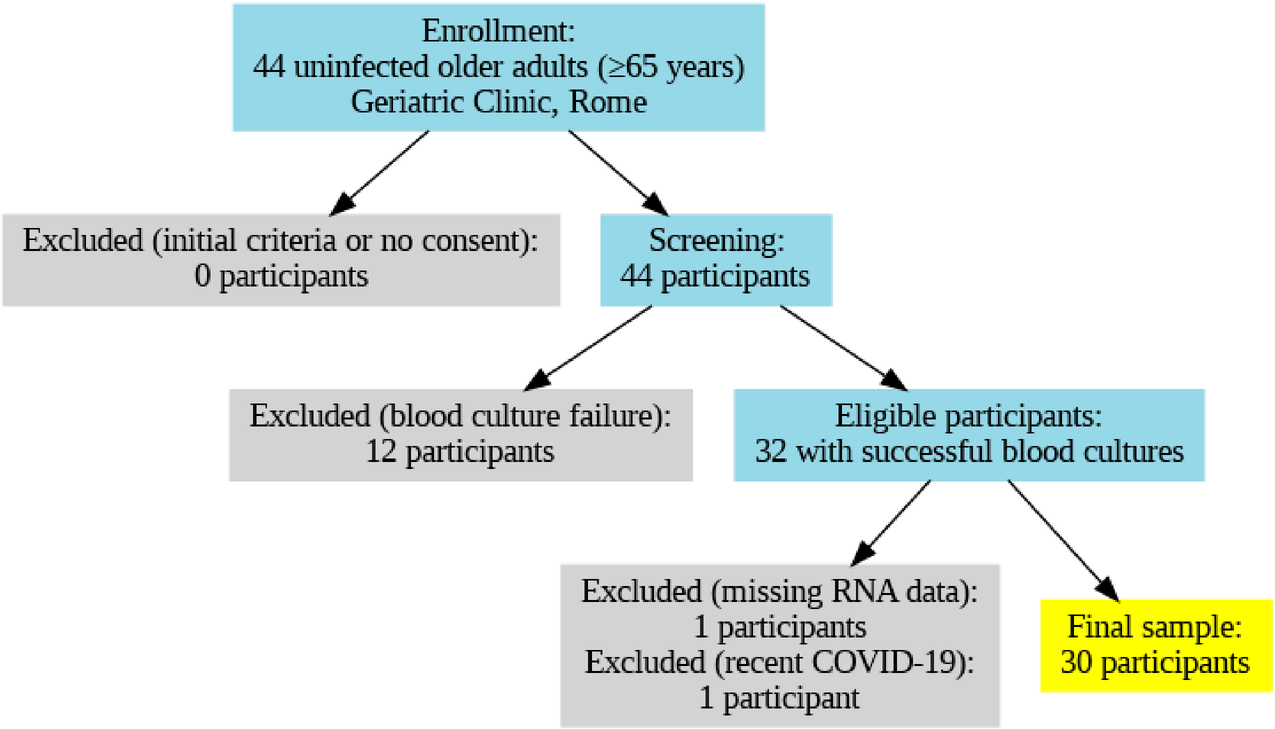

Supplementary Figure S1. Participant enrollment and selection.

Flowchart depicting recruitment, screening, and exclusion criteria for study participants. A total of 44 uninfected older adults (≥ 65 years) were enrolled at the Geriatric Clinic in Rome. Twelve participants were excluded due to blood culture failure. Of the 32 eligible participants with successful blood cultures, one was excluded due to missing RNA data and one due to recent COVID-19 infection, resulting in a final study sample of 30 participants.
